# From genes to folds: a review of cortical gyrification theory

**DOI:** 10.1007/s00429-014-0961-z

**Published:** 2014-12-16

**Authors:** Lisa Ronan, Paul C. Fletcher

**Affiliations:** Brain Mapping Unit, Department of Psychiatry, University of Cambridge, Sir William Hardy Building, Downing Site, Downing Street, Cambridge, CB2 3EB UK

**Keywords:** Gyrification, Morphology, Cerebral cortex, Tangential expansion

## Abstract

Cortical gyrification is not a random process. Instead, the folds that develop are synonymous with the functional organization of the cortex, and form patterns that are remarkably consistent across individuals and even some species. How this happens is not well understood. Although many developmental features and evolutionary adaptations have been proposed as the primary cause of gyrencephaly, it is not evident that gyrification is reducible in this way. In recent years, we have greatly increased our understanding of the multiple factors that influence cortical folding, from the action of genes in health and disease to evolutionary adaptations that characterize distinctions between gyrencephalic and lissencephalic cortices. Nonetheless it is unclear how these factors which influence events at a small-scale synthesize to form the consistent and biologically meaningful large-scale features of sulci and gyri. In this article, we review the empirical evidence which suggests that gyrification is the product of a generalized mechanism, namely the differential expansion of the cortex. By considering the implications of this model, we demonstrate that it is possible to link the fundamental biological components of the cortex to its large-scale pattern-specific morphology and functional organization.

## Introduction: key characteristics of gyrification

A wing would be a most mystifying structure if one did not know that birds flew. One might observe that it could be extended a considerable distance that it had a smooth covering of feathers with conspicuous markings, that it was operated by powerful muscles, and that strength and lightness were prominent features of its construction. These are important facts, but by themselves they do not tell us that birds fly. Yet without knowing this, and without understanding something of the principles of flight, a more detailed examination of the wing itself would probably be unrewarding (Barlow 1961).

Barlow’s eloquent description of the limitations of non-contextualized observations applies well to the current state of our understanding of brain shape. While there have been many intriguing observations, the lack of a satisfactory, over-arching model renders it difficult to interpret the biological meaning of cortical morphology. A central problem is that cortical morphology is characterized and measured at a large scale, while the most important fundamental insights to cortical development occur at the microscopic level. In this paper, we attempt to bridge this gap.

To begin with, it is worth noting that any theory of gyrification must explain certain consistent observations. Of these, the most prominent is the pattern specificity of folds, i.e., folds are strikingly consistent across individuals (and even some species) in terms of position, orientation and the temporal pattern of development (Welker 1990; Borrell and Reillo 2012). This pattern specificity is evident over and above the considerable inter-individual variation in the exact morphology of the folds (White et al. 1997). Furthermore, there is a noted hierarchy to this specificity, where the deepest and the most stable folds—the so-called primary sulci that appear earliest in gestation—are more heritable than secondary and tertiary folds (Lohmann et al. 1999).

Pattern specificity is also found with regard to another key characteristic, namely the co-localisation of folding and underlying cytoarchitecture. In particular, the primary sulci can demonstrate very consistent relationships to the point where cytoarchitectonic boundaries may be reliably associated with specific folding features (Welker 1990). This relationship between macro- and microstructural features becomes more variable for secondary and tertiary sulci (Fischl et al. 2008).

The pattern specificity of folding not only encompasses a range of inter-dependent, hierarchical characteristics, but also compellingly indicates that gyrification is not simply a random mechanical process. Rather the pattern specificity of folds, their co-localization with cytoarchitecture, and their heritability together denote the biological significance of large-scale morphology. Given that the folding of the cortex is a physical process, the biological interpretability of morphology is rooted in our understanding of this mechanism.

## Theories of cortical gyrification

**Fig. 1:**
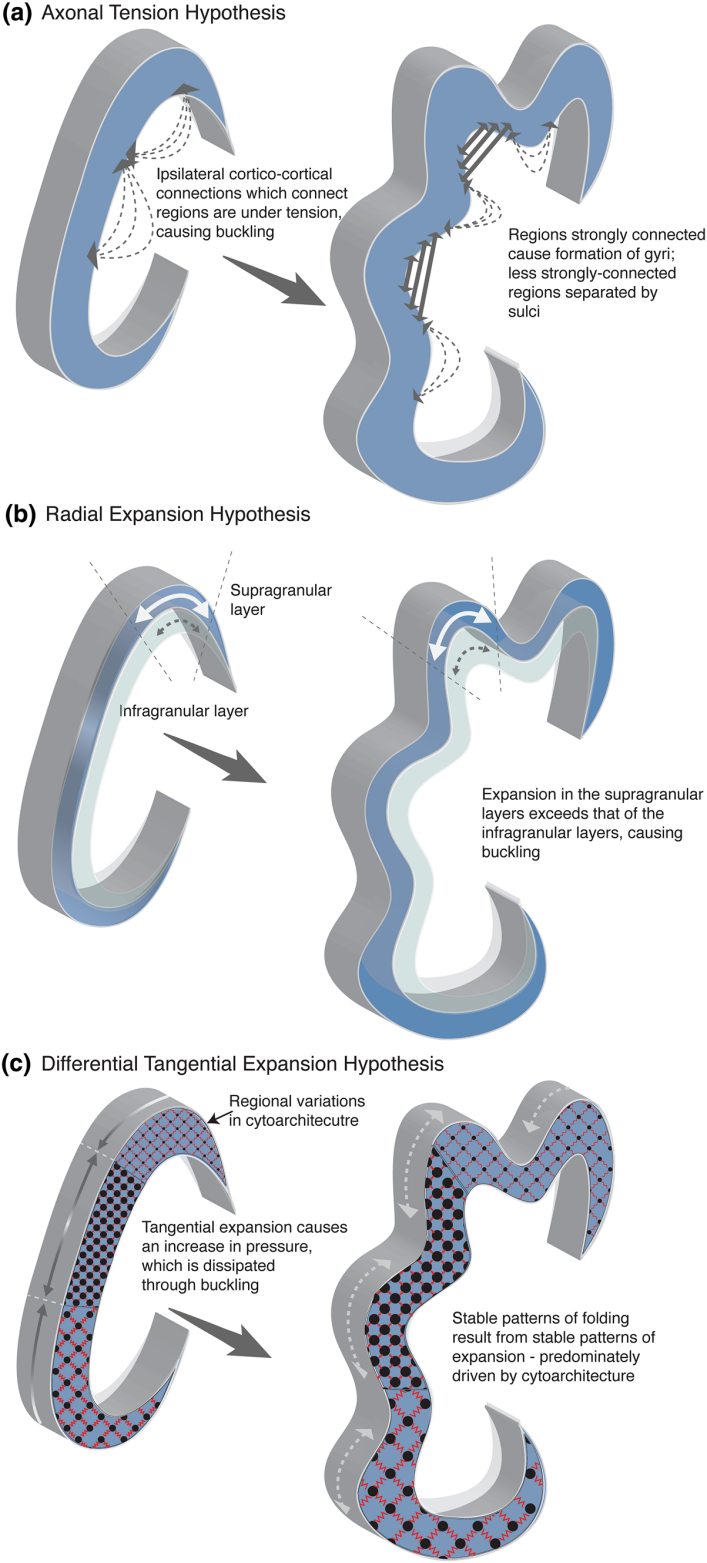
Three distinct mechanisms proposed for gyrification. **a** The axonal tension hypothesis proposes that axons under tension pull regions of the cortex which are strongly connected together, causing folds. However, there are a number of problems with this hypothesis (1) axonal connectivity is not commensurate with the hypothesized pattern of connectivity; (2) axonal innervation post-dates the formation of folds; (3) axons are not under requisite tension to cause folding; (4) removal of axons during developing causes an increase in the number of folds. **b** The radial gradient hypothesis proposes that the increase in expansion of the supragranular layers relative to the infra-granular layers causes buckling. However several experimental observation militate against this (1) the incidence of bRG (which contribute to supragranular layer expansion) is similar in gyrencephalic and lissencephalic species; (2) gyrification may be induced without a change in the proliferation of bRG; (3) reduction in the proliferation of bRG does not change the degree of gyrification; (4) disruption in the formation of supragranular layer neurons does not affect gyrification. **c** The differential tangential expansion hypothesis proposes that tangential expansion of the cortex causes an increase in tangential pressure which is mitigated though buckling. Empirical evidence suggests that the pattern of differential expansion (predominantly influenced by the pattern of cytoarchitecture), causes pattern-specific folding. As such, the stability of folds represents the stability of expansion forces in that region

Gyrification may be considered from two distinct though related perspectives: namely, the nature of the force that causes the surface to buckle and, separately, the factors which mediate this buckling to cause pattern-specific folding. To date, many different mechanisms have been proposed as the primary force driving gyrification. One of the most prominent is the axonal tension theory, which postulates that axons “pull” on the cortex, forming gyri and by geometric necessity sulci also (Van Essen 1997) (see Fig. 1). However, recent investigations militate against this hypothesis (Xu et al. 2010; Ronan et al. 2013; Sun and Hevner 2014; Taber 2014). It has also been proposed that the limiting volume of the cranium causes the expanding cortex to crumple. However, empirical investigations out-ruled this theory (Barron 1950). Another line of thought is that folds arise due to surface expansion, which engenders a pressure within the surface that is subsequently mitigated through folding. The exact nature of these forces has, however, been disputed. One suggestion is that the relative increase in surface expansion of the supragranular (upper) layers of the cortex relative to the infra-granular (lower) layers causes surface folding (Richman et al. 1975) (see Fig. 1). Alternatively, it has been proposed that it is the tangential surface expansion that gives rise to in-plane pressure which is dissipated by out-of-plane folding (Le Gros Clark 1945; Ronan et al. 2013) (see Fig. 1).

### Cortical expansion and gyrification

**Fig. 2:**
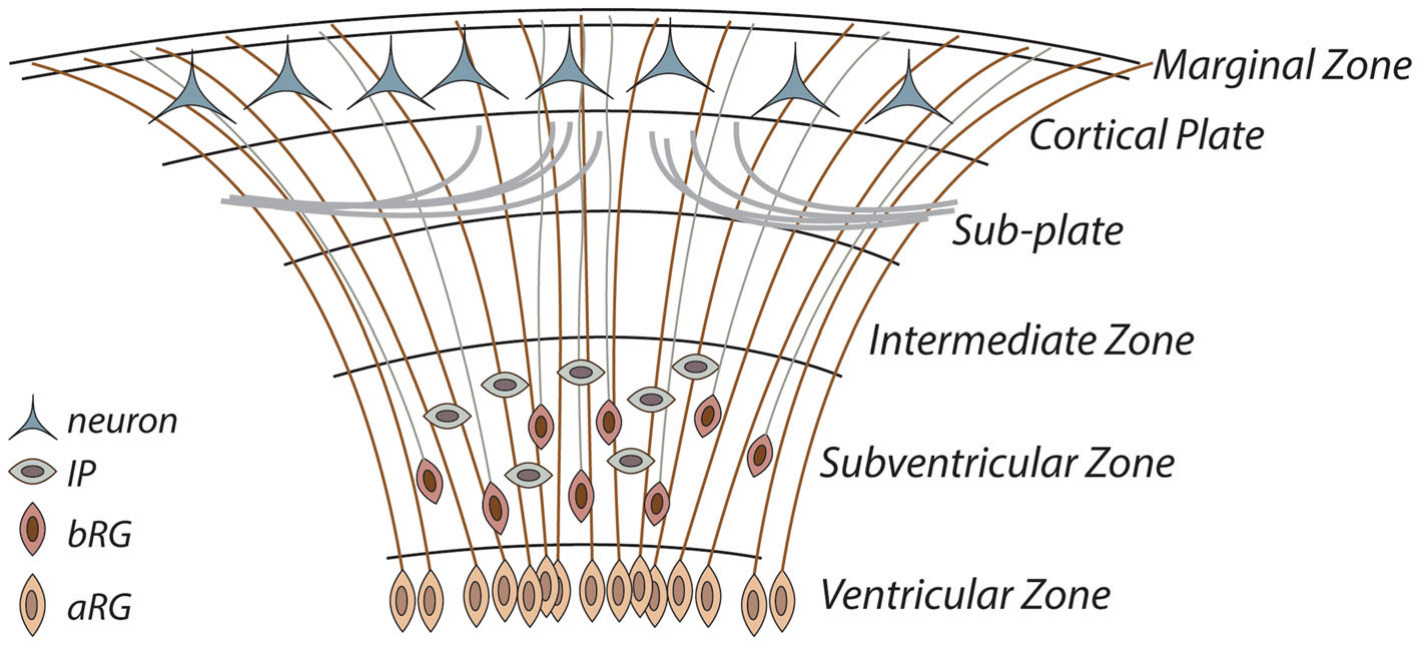
Developmental neurogenesis is driven by apical radial glia (aRG) in the ventricular zone, and intermediate progenitor cells (IPCs) and basal radial glia (bRG) in the sub-ventricular zone

The relative merits of each of the expansion hypotheses may be more fully appreciated given a brief outline of our current understanding of cortical development (Fig. 2). This is fundamentally based on the radial unit hypothesis which postulates that development begins with a period of symmetric division of cells along the ventricular wall. This dramatically increases the number of founder progenitor cells (neuroepithelial cells and radial glia), which directly and indirectly give rise to the neurons of the cortex (Rakic 1995). At the onset of neurogenesis, these so-called apical progenitor cells (Dehay and Kennedy 2007; Fietz and Huttner 2011) divide asymmetrically producing either a neuron or two other types of progenitor cells, namely basal radial glia (bRG) or intermediate progenitor cells (IPCs). The neurons derived here migrate along the radial glia to form the infra-granular layers of the cortex, while the daughter progenitor cells (so-called basal progenitor cells) translocate to a more basal layer called the sub-ventricular zone (SVZ), which is characterized by two distinct lamina called the inner SVZ (ISVZ) and the outer SVZ (OSVZ), respectively. In primates, IPCs and bRG undergo several rounds of symmetric division to produce neurons (Betizeau et al. 2013). The development of the cortex proceeds in an inside–out fashion, with neurons destined for lower cortical layers generated first (primarily in the VZ), while upper cortical layers neurons are generated later (generally in the SVZ), and migrate past cells generated earlier to populate increasingly superficial positions in the cortex (Dehay and Kennedy 2007; Betizeau et al. 2013; Geschwind and Rakic 2013). It has been demonstrated that the migration of all neurons follows a conical trajectory which acts to increase the tangential spread of neurons across the early developing cortical plate prior to gyrogenesis (Torii et al. 2009; Reillo et al. 2011; Borrell and Reillo 2012). This conical spread increases exponentially for supragranular neurons generated in the SVZ, further enhancing the tangential expansion of the cortex in species with enlarged SVZ layers and increased cell proliferation in these layers. Finally, axons innervate the cortical plate after a prolonged waiting period in the sub-plate, the transient substrate of the cortical plate (Kostovic and Rakic 1990).

Much of this knowledge has come from investigations into evolutionary changes of the cortex, in particular focusing on comparisons between gyrencephalic and lissencephalic species. For the purposes of gyrification theory, there are a number of relevant points. First, the duration of mitosis and neurogenesis in the VZ is linked to the degree of gyrification, with human neurogenesis lasting almost twice as long as that in macaques (Rakic 1995). Disruptions to mitosis, whether by genetic manipulation of β-catenin (which controls the number of cells in cycle (Chenn and Walsh 2002), or of caspase which controls apoptosis (Haydar et al. 1999), have a predicted effect on the degree of folding, and can be used to contrive gyrification in otherwise lissencephalic cortices.

Secondly, it is noted that the presence of bRG and IPCs in the OSVZ greatly increases the tangential expansion of the cortex in gyrencephalic species (Reillo et al. 2011; Betizeau et al. 2013) and is correlated with the degree of gyrification (Pilz et al. 2013; Reillo et al. 2011). Given that the neurons generated in the SVZ predominantly populate the upper layers of the cortex, this observation seems to support the hypothesis that gyrification may be the result of an increase in supra- vs. infra-granular layer expansion (Richman et al. 1975). However, other studies contrasting different species demonstrate that the presence and incidence of bRG is similar in some gyrencephalic and lissencephalic species (Garcia-Moreno et al. 2012; Kelava et al. 2012), militating against this hypothesis. More directly, manipulations to limit supragranular layer neurogenesis (predominantly layer 2) in the gyrencephalic cortex of the ferret do not disrupt the normal degree and pattern of gyrification (Poluch and Juliano 2013). Moreover, manipulation of neurogenesis in the VZ, without a concomitant increase in the number of bRG, has been used to induce gyrification in the otherwise lissencephalic cortex of the mouse (Rash et al. 2013). Taken together these results militate against the hypothesis that the radial gradient of tangential expansion between layers in the cortex is the primary mechanism of gyrification, though it may augment folds already formed. Instead, the evidence suggests that gyrification is primarily a function of the overall tangential expansion of the cortex to which these cells contribute.

In summary, two important points are evident. In the first instance, gyrification is primarily driven by the tangential expansion of the developing cortex. Mechanically, it is hypothesized that folding mitigates the resulting increase of pressure within the surface (Le Gros Clark 1945; Ronan et al. 2013) (see Fig. 1). The second important point is that a number of factors contribute to tangential cortical expansion. As discussed above, the evolutionary, order-specific increase in cortical expansion may be attributable to a number of separate mechanisms including prolonged neurogenesis, the increase in number and type of progenitor cells and the conical migration trajectories of neurons to the developing cortex. While these adaptations have been demonstrated to increase gyrification, no single one has been identified as unique to gyrencephalic species (Borrell and Reillo 2012). For example, the impact of bRG on cortical gyrification is heterogeneous with some studies indicating a correlation of the presence and incidence of bRG with gyrification, while other studies do not. Similarly, while some studies indicate that manipulation of bRG can contrive or alter gyrification (Stahl et al. 2013; Reillo et al. 2011), other studies (in other species) fail to demonstrate such changes (Rash et al. 2013; Poluch and Juliano 2013). In and of themselves, these studies are not contradictory being carried out for different species, in different parts of the brain at different points in development (Nonaka-Kinoshita et al. 2013). Rather, the variable impact of the presence and incidence of bRG and its relation to gyrification serve to illustrate a more general point which is that gyrification is the generalized result of tangential cortical expansion which is itself influenced by multiple factors, which act to different degrees in different species.

The implication of these observations is that gyrencephaly is not reducible to a single evolutionary adaptation, but rather is the generalized mechanical product of the tangential expansion which itself is a function of multiple developmental processes. This, in turn, may explain the independent occurrence of gyrencephaly across mammalian orders (Lui et al. 2011; Borrell and Reillo 2012) as well as the noted deviations of certain species from expected linear trends (Hofman 1989; Zilles et al. 2013).

### Genes and gyrification

This view of gyrification as the aggregate of multiple factors which contribute to surface expansion fits with observations of how genes and transcription factors (TFs) variously induce morphological abnormalities. These have been extensively reviewed elsewhere (Hevner 2006), but point to the general principle that those factors which promote surface expansion through an increase in progenitor proliferation (in particular proliferation of radial glia) result in an increase in surface expansion and hence gyrification (Chenn and Walsh 2002). For example, FGF2, the manipulation of which can be used to induce folding, promotes RG self-renewal leading to an increase in tangential cortical expansion (Rash et al. 2013). Other factors which prevent apoptosis may be used to artificially maintain the progenitor pool, similarly increasing expansion (Haydar et al. 1999). On the other hand, genes which promote neuronal differentiation (thus depleting the progenitor pool), cell apoptosis or radial migration attenuate surface expansion and hence gyrification. Some of these have been linked to specific diseases characterized by abnormal gyrification (Mochida and Walsh 2001). These include LIS1 and DCX implicated in radial migration and linked to lissencephaly (Sapir et al. 1999; Taylor et al. 2000; Borrell and Reillo 2012). Also noteworthy is ASPM, linked to reduced surface area and simplified gyral patterns (Bond et al. 2002), EMX2 a transcription factor implicated in progenitor proliferation (Galli et al. 2002) and linked to schizencephaly (Walsh 1999) and Gpr56 linked to factors controlling migration (Li et al. 2008), and frontal lobe polymicrogyria (PMG) (Piao et al. 2004).

In short, factors that act to increase the tangential expansion of the cortex (such as evolutionary adaptations of cell type, genes which increase proliferation or the divergent trajectory of migrating neurons) result in an increased degree of gyrification, while factors that decrease expansion (such as reducing radial migration and proliferation potential of cells) decrease gyrification (Kriegstein et al. 2006; Lui et al. 2011; Reillo et al. 2011). However, expansion alone is not sufficient to cause folding as evidenced by the fact that some cortices are lissencephalic despite undergoing developmental expansion. In the next section, we will consider the additional requirements necessary to cause folding, and how these factors give rise to pattern-specific folding.

## Pattern-specific folding

As discussed previously, cortical gyrification is not simply folding, but rather *pattern*-*specific* folding. By adopting tangential expansion as the primary mechanism of gyrification, we can in turn consider the factors which mediate this process to produce characteristic features of sulci and gyri.

In fact, the phenomenon of pattern-specific folding is directly implied by the tangential expansion model of gyrification and simply related to the fact that cortical expansion is non-uniform. Mechanically, this means that the tangential folding forces in the cortex are also non-uniform and result in non-uniform folding, which is observed. If the pattern of non-uniform expansion is consistent across individuals, it follows that the pattern of folding will be also. We consider this argument in more detail below.

### Non-uniform cortical expansion

In the developing cortex, several factors contribute to the pattern-specific, non-uniform (or differential) cortical expansion. Initially, regional expansion is controlled by mitosis and governed by the protomap (Rakic et al. 2009). Once neurogenesis has completed, surface expansion is driven by cellular growth, differentiation and apoptosis, and the growth and formation of connections. Collectively, these factors are ultimately reflected in regional cytoarchitecture. Given this, it is the case that cytoarchitecture is causally linked to regional expansion, which in turn is causally linked to gyrification. Therefore, cytoarchitecture and gyrification are linked via the mechanism of regional expansion. It follows that if cytoarchitecture (which reflects regional expansion) has a broadly consistent pattern across the cortex (which is observed), we will also observe a broadly consistent pattern of folding. Put another way, the differential expansion model of pattern-specific gyrification, suggests that the pattern specificity of folding is related to the pattern specificity of regional expansion, which may be related to the pattern specificity of cytoarchitecture. The validity of this model of the origin of pattern-specific folding is most convincingly demonstrated by enucleation experiments.

Enucleation is the removal of the eyes of a developing fetus, which in turn results in the specific reduction of axonal connectivity from the lateral geniculate nucleus to the striate cortex. In a series of experiments, the effects of enucleation were contrasted between a so-called period of “early-enucleation” in the first half of gestation, prior to the innervation of thalamo-cortical axons, and “late enucleation” in the second half of gestation and after innervation. The results of the enucleation experiment were surprising. Following late enucleation, the normal patterns of cytoarchitecture and gyrification were preserved; however, in the early enucleates, there were considerable changes both in the extent of the primary visual cortex which was reduced by 70 % (Dehay et al. 1991), and the degree of cortical gyrification which was significantly increased (Rakic 1988).

The importance of these results is twofold. In the first instance, these experiments confirmed the importance of thalamo-cortical innervation for the appropriate formation of cytoarchitectonic boundaries. However, they also provided a significant test for the relationship between pattern-specific gyrification and cytoarchitecture by directly examining the effect of cortical arealization (i.e., the pattern of cytoarchitecture) on the pattern specificity of folding. As outlined above, if the pattern of cytoarchitecture (size, position, etc.) is abnormal, it will reflect an altered pattern of expansion and hence folding (which was observed in the early enucleates). On the other hand, if the pattern of cytoarchitecture is normal, then so too should the pattern of folding (observed in the late enucleates). One subtlety of these experiments is that although the pattern of folding in the early enucleates was abnormal, it was nonetheless repeatable across animals (Rakic 1988), further supporting the hypothesis that the pattern of expansion is the generalized mechanism controlling pattern-specific folding.

An additional critical point is as follows: the overall surface area of the occipital–temporal cortex in the early enucleates was not changed, though there was an increase in the degree of gyrification (Dehay et al. 1996). This is consistent with our suggestion that it is not the total expansion of the cortex, but rather its differential expansion (i.e., differences in regional expansion), that affects the pattern of folding. Such a relationship has been illustrated elsewhere and by different mechanisms. For example, making use of the fact that different regions of the cortex develop at different rates, Poluch and Juliano (2013) were able to selectively reduce layer 4 neurogenesis and hence expansion of the parietal but not the temporal lobe in the ferret. In normal development, the parietal lobe exceeds the temporal cortex in terms of expansion and folding. However, after the manipulation attenuating parietal expansion, there was a loss in gyrification relative to the unchanged temporal lobe. These results confirm that the pattern of gyrification (position, orientation and degree of folds) is a function of the differential expansion of cortex which engenders predictable, non-uniform tangential pressures resulting in broadly consistent cortical morphology. Moreover, by linking regional expansion (driven by the intrinsic architecture of the cortex) to pattern-specific folding, we are able to accommodate another key characteristic of gyrification, namely the co-localisation of folds and cytoarchitecture (Welker 1990; Fischl et al. 2008). This relationship may also explain in part the increasing degree of gyrification associated with increasing degrees of arealization observed across multiple species (Welker 1990), as well as the differences between orders in terms of the pattern and degree of folds (Zilles et al. 2013).

In summary, we have argued that empirical evidence from multiple sources suggests that cortical gyrification is primarily the result of mechanical buckling of the cortex owing to an increase in tangential pressure due to surface expansion but that this model is by itself not enough to explain the pattern specificity of gyrification. Instead, we suggest that differential expansion (i.e., variations in the degree of local expansion) will result in differential folding forces leading to non-uniform folds. If the pattern of differential expansion is consistent, then it follows that the pattern of folds will likewise be broadly consistent. Mathematical models support this hypothesis (Toro and Burnod 2005; Tallinen et al. 2014).

The results of the enucleation experiments suggest that the emergence of sulci and gyri cannot be divorced from thalamo-cortical and cortico-cortical connectivity, and that axons contribute significantly to the formation of pattern-specific folding. However, they do so by controlling regional maturation (Dehay et al. 1991; O’Leary et al. 2007), and not by exerting mechanical forces as has previously been postulated (Van Essen 1997).

### Pattern-specific folding: further considerations

A number of other factors are related to the emergence of specific folding features via the mechanism of differential expansion. Beginning with the earliest in terms of development, it is known that mitosis in the embryonic brain is region specific (Dehay et al. 1993). For example in the ferret, mitosis in the VZ and OSVZ is 1.4 times greater in prospective splenial gyrus than in prospective lateral sulcus (Reillo et al. 2011). Such variations are linked to region-specific differences in expansion. In humans, where the parietal and temporal cortex have increased expansion and folding compared to the insula and cingulate, there is a twofold increase in the density of proliferative progenitors in the OSVZ in the former regions (Reillo et al. 2011). As such, progenitors which contribute to surface expansion accumulate to a greater extent in regions that undergo greater degrees of expansion, and have been observed to vary in a manner predictive of the formation of sulci and gyri (Smart et al. 2002; Bayer and Altman 2006; Kriegstein et al. 2006). These variations may contribute to region-specific tangential expansion, resulting in predictable patterns of tangential forces and hence folding. Biomechanical feedback processes may also contribute to this process and augment early subtle distinctions, further enhancing folding patterns.

It has also been observed that regional differences in pre-plate axonal innervation co-vary with the pattern of cortical folds (Kostovic and Rakic 1990). In and of themselves these variations do not constitute a mechanism of gyrification. However, under the force of expansion, these sites may represent points of maximal/minimal resistance to tangential folding forces, and in turn facilitate the formation of folds commensurate with the pattern of these early variations. In a similar way, the variable thickness of the transient layers which contribute to cortical development, may additionally influence the position of folds.

### The scale of folding forces

In short, multiple factors may mediate tangential expansion and contribute to the pattern specificity of folds. A critical point is that regional variations in expansion may be considered to occur at multiple scales. For example, as well as pro-gyral/pro-sulcal differences already detailed above (Smart et al. 2002; Bayer and Altman 2006; Kriegstein et al. 2006; Rajagopalan et al. 2011), different degrees of neuronal spacing have been observed at the cytoarchitectonic level (Semendeferi et al. 2011), while at a larger scale still, there is a rostral–caudal gradient in development (Smart et al. 2002). Importantly, the fact that folding occurs at a scale much greater than the scale of neurons and connections which fundamentally drive expansion indicates that tangential pressure builds up over many scales, and ultimately aggregates at a large scale to cause folding. This makes sense when one considers that the force of a single neuron/group of neurons is negligible, but taken as an aggregate across the cortex sums to a magnitude sufficient to drive the expansion and folding the cortex as previously discussed. Another implication is that the distance over which these folding forces act may be different for each fold. For example, if there is a marked difference in regional expansion at the cytoarchitectural level (i.e., between two neighboring regions, or between a pro-gyral region vs. a pro-sulcal region), then the local expansion forces may be large enough to result in a fold. If on the other hand, neighboring regions, though cytoarchitecturally distinct, are not different enough to engender a large differential, then the folding force may instead aggregate over a larger area (e.g., it may be that the central sulcus emerges due to the rostral–caudal gradient of development rather than because of differences in cytoarchitecture in that area).

Ultimately, the pattern of folding forces is determined by the intrinsic architecture of the cortex, and the size, relative position and temporal maturation of distinct cortical areas. Variations in each of these characteristics will give rise to a unique pattern of differential expansion and in turn the unique morphology of each individual, as well as the variable co-localization between cytoarchitectonic boarders and specific folding features. Regions which have the most stable patterns of expansion will also have the most consistent co-localization between cytoarchitectonic boarders and cortical morphology, while more variable patterns of expansion will result in a more variable relationship. This view of the origin of pattern-specific folding may also explain why we observe decreasing consistency in position and morphology of secondary and tertiary sulci, given that these latter folds emerge in the context of more stable, primary folds. In a similar way, this may also explain the relative conservation of folding patterns observed across a number of species (Borrell and Reillo 2012). For example, it is observed that patterns of cytoarchitectural organization are largely consistent (e.g., in mammals motor and somatosensory regions always lie adjacent to each other), while larger brains with more complex morphology tend to exhibit additional, newer cortical areas (Welker 1990).

## Summary

There has been a significant advance in recent years in the understanding of factors which affect the development of the cortex and the onset of gyrification (Kriegstein et al. 2006; Lui et al. 2011; Fietz and Huttner 2011; Borrell and Reillo 2012; LaMonica et al. 2012). However, these empirical investigations, while critical to understanding the generalized nature of gyrification as a function of cortical expansion, do not explicitly address the physical mechanism which engenders folding. In this manuscript, we argue that multiple strands of evidence suggest that cortical gyrification is primarily driven by the tangential expansion of the cortex. While many models of gyrification have indicated the importance of an accurate representation of underlying white matter, it is nonetheless the expansion of the cortex that induces folding forces (Tallinen et al. 2014; Toro and Burnod 2005). We hypothesize that folds occur to mitigate the increase in pressure arising from surface expansion. As an extension of this, the pattern specificity of folds arises from the pattern specificity of expansion which is driven at the smallest level by the proliferation and growth of cells and their connections which are regionally distinct. An important implication of this model is that gyrencephaly is a generalized mechanical product of differential tangential surface expansion and is not reducible to a single evolutionary adaptation.

As well as providing a framework to contextualize the role of various genetic and developmental factors on gyrification, a mechanistic account of folding is critical to the biological interpretation of cortical morphology. For example, under the ageis of the axonal tension hypothesis, sulci and gyri are hypothesized to arise from and hence reflect ipsilateral-cortico-cortical connectivity (Van Essen 1997). However, if sulci and gyri arise from the differential expansion of the cortex driven by it intrinsic architecture, then such an interpretation is invalid. Instead, morphological parameters sensitive to the intrinsic nature of the surface will offer greater sensitivity to differences in the mechanism of folding as well as increased biological interpretability (Ronan et al. 2011, 2012, 2013).
